# Factors associated with phosphate homeostasis in children with beta-thalassemia major: An analytical cross sectional study from Pakistan

**DOI:** 10.1371/journal.pone.0316566

**Published:** 2025-02-21

**Authors:** Lena Jafri, Arsala Jameel Farooqui, Bushra Moiz, Aisha Sheikh, Hafsa Majid, Sarah Nadeem, Ruhul Quddus, Samia Khan, Qurat-ul-Ain Khan, Aysha Habib Khan

**Affiliations:** 1 Section of Chemical Pathology, Department of Pathology & Laboratory Medicine Aga Khan University (AKU), Karachi, Pakistan; 2 Department of Pathology and Laboratory Medicine Aga Khan University, Karachi, Pakistan; 3 Department of Medicine, Aga Khan University, Karachi, Pakistan; 4 Endocrinologist, Kelsey-Seybold Clinic, Houston, Texas, United States of America; 5 Fatimid Foundation Karachi, Karachi, Pakistan; Al Muthanna University, IRAQ

## Abstract

**Introduction:**

Children with beta-thalassemia major (β-TM) commonly experience metabolic bone diseases. Understanding fibroblast growth factor 23 (FGF-23) levels in these children can shed light on phosphate dysregulation. This study aimed to assess changes in phosphate homeostasis and associated factors, including FGF-23 and explore relationships between iron overload, FGF23 levels, and phosphorus regulation for clinical management of phosphate disorders, in children with β-TM.

**Methods:**

143 β-TM patients (57.3% male, median age 12 years) were recruited from Fatimid Foundation Karachi, a blood transfusion facility from January to October 2022. Clinical and biochemical evaluations were conducted at Aga Khan University Hospital, including serum ferritin, calcium (Ca), phosphate (P), vitamin D levels, and FGF-23. Descriptive and inferential statistics including multivariable analysis were applied.

**Results:**

This study enrolled 143 patients, with 57.3% males. The median age was 12 years, with 53% underweight. Blood transfusion rates varied, with 66.4% receiving 2/month. Bone/joint pain was reported by 76.2%, with 60.8% requiring analgesics. Median serum ferritin was 2768.3 ng/mL. Hypophosphatemia and hyperphosphatemia were observed in 5.6% and 3.5% of participants, respectively. Vitamin D deficiency/insufficiency affected 92.3%. Plasma c-FGF23 was elevated in 60.8%, while i-FGF23 was high in 14%. A low TMP-GFR (glomerular filtration rate) was associated with high c-FGF23 and low i-FGF23. Multivariable regression revealed c-FGF23, TMP:GFR, Corrected Ca, iPTH, and an interaction term between corrected Ca and iPTH as predictors of serum P variability (~75%).

**Conclusion:**

The study identified contributors to the variations observed in serum P levels in individuals with β-TM and recommends multidisciplinary care and prospective future studies to form targeted interventions for this population.

## Introduction

Beta-thalassemia major (β-TM) is an autosomal recessive blood disorder requiring regular blood transfusions and iron (Fe) chelation therapy for survival since early childhood [1]. In Pakistan every year a range of 4,000 to 9,000 newborns with β-TM are projected to be included in the affected population. Considering a birth rate of 1.3 per 1,000 live births, it is estimated that around 5,250 infants diagnosed with β-TM are born in Pakistan each year [2]. The carrier frequency ranges from 5–8% and it is found in people of all ethnicities [3–5]. Patients with β-TM are prone to growth retardation, bone pains, and fragility fractures [6–8] secondary to excessive erythropoiesis, hormonal imbalances, bone hypoxia, hemochromatosis, and chelation therapy [9–11]. Iron and P metabolism must be properly regulated to sustain optimal bone formation. In the last decade, researchers have focused on abnormal calcium (Ca) homeostasis and vitamin D deficiency in thalassemia patients, but phosphate (P) homoeostasis has received little attention [12–16]. There is evidence of both hypo- [17, 18] and hyper-phosphatemia in patients with β-TM in literature [5, 19].

Under physiological conditions, intestinal absorption, bone deposition, and renal excretion are tightly regulated to maintain the serum P concentration within normal range. Fibroblast growth factor 23 (FGF-23) plays a pivotal role in maintaining P homeostasis by regulating both P metabolism and levels of vitamin D. Primarily synthesized by osteocytes and osteoblasts within bone tissue, FGF23 serves to lower serum P levels through two main mechanisms: firstly, by inhibiting P reabsorption in the kidneys, and secondly, by suppressing the production of 1,25-dihydroxy vitamin D (1,25 (OH)_2_D), which would otherwise enhance the absorption of P. FGF23 inhibits the activity of 1 alpha hydroxylase, thereby reducing the production of (1,25 (OH)_2_D) by diminishing the messenger RNA level of Cyp27b1, which is essential for converting 25-hydroxyvitamin D (25OHD) into its active form, (1,25 (OH)_2_D). As a result, FGF23 plays a crucial role in maintaining the balance of P within the body, preventing the onset of hyperphosphatemia, and ensuring optimal bone mineralization. Additionally, FGF23 stimulates the synthesis of Cyp24a1, which degrades 1,25 (OH)_2_D. Another effect of FGF23 is its regulation of NaPi-2b expression in the small intestine. Hypophosphatemia, diminished levels of 1,25 (OH)_2_D, elevated plasma intact parathyroid hormone levels (iPTH), and compromised mineralization of bone and cartilage are outcomes of excessive FGF23 production.

An excess of Fe can disturb the FGF23-P equilibrium in β-TM, resulting in degenerative abnormalities in the skeletal system. The precise mechanism by which the interaction between iPTH and FGF23 regulates serum P remains unclear. Several recent studies have indicated that serum Fe and excessive Fe accumulation can influence serum P levels [20–22]. However, there is insufficient data available to thoroughly assess the association or interaction between P, FGF23 and other biochemical biomarkers in children with β-TM [23].

The objective of the study was to assess the alterations in phosphate homeostasis and its associated factors, including the phosphaturic hormone FGF23, in children with β-TM. Additionally, this study aimed to examine the potential relationships between iron overload, FGF23 levels, and phosphorus regulation, highlighting the implications for clinical management of phosphate disorders in this population

## Materials and methods

### Study setting

From August 2021 to June 2023, an observational cross-sectional descriptive study was conducted in collaboration with Fatimid Foundation Karachi (FFK) at the Sections of Clinical Chemistry & Haematology, Department of Pathology and Laboratory Medicine, and Department of Medicine, Aga Khan University (AKU), Pakistan. The participants were recruited from FFK, a non-profit organisation that provides free blood components to patients suffering from various chronic blood disorders between 09/01/2022 until 02/10/2022. The Section of Clinical Chemistry, Department of Pathology and Laboratory Medicine, AKU, performed all biochemical analyses.

### Sample size calculation

Given the fixed population size of 24,000 registered individuals with β-TM in Pakistan and an annual projected increase of 5000 cases [2], the following calculation was employed to determine the necessary sample size: with a 95% confidence interval, 3.2% margin of error (d), and an expected prevalence of iron overload (p) of 10.3%, a sample size of 142 children was required for this study. To address the possibility of analytical error (12%), a sample size of 152 children with β-TM was targeted [24].

### Patients recruitment & eligibility criteria

Patients with β-TM were recruited using convenient sampling, in which consecutive patients who met the eligibility criteria were enrolled until the sample size was reached. Children aged 4 to 18 years who had been diagnosed with transfusion dependent β-TM based on electrophoresis/ HPLC/ molecular studies were eligible for the study. Patients were excluded from the study if they had a blood disorder other than β-TM, were on steroid medication, or had any other unstable illness that precluded participation in the study. Patients with chronic kidney disease (diagnosed using serum Cr-based estimated glomerular filtration rate as a marker of kidney function using the Schwartz formula: eGFR 30 ml/min was used as the cutoff for CKD) were also ineligible. One hundred and fifty-two children with transfusion dependent β-TM were recruited after informed consent.

### Data collection

Clinical information (blood transfusion history, iron chelation), anthropometric characteristics, and a physical examination were obtained from medical charts. A trained research assistant (RA) collected demographic information including ethnicity from patients and/or their guardians using a predetermined questionnaire. Ethnicities were based on self-classification and were Punjabi, Sindhi, Baloch, Pashtuns, Urdu-speaking and other minorities. Participants’ height and weight were measured through stadiometer and calibrated scales and body mass index (BMI) percentiles and Body Surface Area (BSA) (BSA = height cm x weight kg ÷3600) were computed. As per WHO classification patients were classified as underweight (less than the 5th percentile), healthy weight range (5th percentile up to the 85th percentile), overweight (85th to less than the 95th percentile) and obese (equal to or greater than the 95th percentile). The Wong-Baker Faces Pain Rating Scale was used to assess bone pains.

### Blood and urine collection

Fasting blood samples were collected in gel and EDTA tubes by trained phlebotomists at AKUH’s phlebotomy centre close to FFK. Blood was drawn before blood transfusion to avoid biochemical changes due to recent transfusion. Three to five millilitres of random urine samples were also collected. All the samples were centrifuged, aliquoted and refrigerated at 2 to 8 degrees centigrade except PTH (plasma separated and frozen at -20°Cat phlebotomy centres) until transported to the Section of Clinical Chemistry at AKUH in dry ice. The patients same day pretransfusion haemoglobin (Hb) and hematocrit (Hct) levels, analyzed at FFK were recorded from their files.

### Biochemical analysis and urinary indices calculation

Serum Ca, P, Cr, PTH, Mg, and ferritin levels, as well as urinary Ca, P, and Cr, were measured using kits from Siemens USA on ADVIA 1800. Diasorin kits were used to analyse 25OHD and 1,25(OH)_2_D on the LIAISON analyser. c-FGF23 and i-FGF23 were measured using ELISA kits from Immutopics (kits #60–6100 and 60–6600, respectively). For iFGF23, the cutoff was 61.21 pg/ml, while for cFGF23, the cutoff was 33 RU/ml as low and >145 RU/ml as high.

Fractional Tubular Reabsorption of Phosphate (TRP), which is the fraction of P in the glomerular filtrate that is reabsorbed in the renal tubules, was calculated as follows: TRP = 1-[(uP/pP)x(pCr/pCr)]. If TRP is low in hypophosphatemia this usually indicates a renal tubule defect.

The tubular maximum reabsorption of P (TmP: GFR) which is the ratio of tubular maximum reabsorption of P (TmP) to glomerular filtration rate (GFR) was used to evaluate renal P transport. Low levels suggest renal phosphate wasting and is more sensitive as compared to TRP. TmP, GFR was calculated as follows:

If TRP is less than 0.86, then TmP: GFR = TRP x P, and if TRP was greater than 0.86, then: TmP: GFR = [0.3xTRP/1-(0.8xTRP)]c = x P.

All biochemical findings were communicated to the parents or guardian of the children as per study protocol. For patients who showed vitamin D deficiency (25OHD <20 ng/ml) or insufficiency (25OHD 20–30 ng/ml), oral supplements of 600,000 IU cholecalciferol were administered by the RA as per approved study protocol.

### Ethical declaration

Ethical clearance was obtained from the Ethical Review Committees (ERC) of both AKU and FFK, (ERC-FF-03, ERC# 2021-6446-18701) and patients/parents were enrolled after informed consent or assent. Informed written assent or consent was taken from the study participants, depending on their age. For participants under 18 years, the informed consent of the legally authorized representative accompanying them was also obtained. The study purpose was explained to them in the private consultation room at the study setting when they voluntarily provided permission to engage in dialogue with the RA. The procedure was witnessed by an independent third party, whose signatures were also obtained on the forms. For individuals who did not know how to read or write, thumb impressions were obtained on the form. A copy of the signed assent/consent form was given to all participants.

Digital anonymity was ensured throughout study by using anonymized participant IDs in the data files. The data files were only available to authorized research staff, and the hard copies of the forms stayed with the principal investigator. The data will be stored for a total of seven years, according to institutional guidelines after which it will be destroyed.

### Statistical analysis

Statistical analysis was done using SPSS software (IBM SPSS, Statistics for Windows, USA) version 19.0 and Stata 17.0. For continuous variables, descriptive statistics included mean, standard deviation, and median (IQR), and for categorical variables, frequencies and percentages were reported. For inferential statistics, we compared the means and medians of biochemical parameters in male and female participant groups using an independent sample Student’s t-test and Mann-Whitney U test, respectively. Spearman correlations were reported for skewed continuous variables that displayed a monotonic relationship. The factors affecting serum P levels were investigated using backward stepwise multiple linear logistic regressions. For multivariable analysis, we conducted backward stepwise regression to reduce suppression effect, and all the probable predictor variables were introduced into our multivariable model. One by one, the predictors with no significant association with P were removed. Statistical significance was considered as a p value < 0.05.

## Results

### Demographics

A total of 152 individuals were recruited for data collection. However, 4 participants were excluded due to an inability to give urine samples, and 5 participants were excluded for failing to provide adequate plasma samples for analysis, leaving 143 participants in the final analysis. A total of 143 participants (82 males and 61females) with a median (IQR 1–3) age of 12 (10–17) years were included in the final analysis. The age groups were divided into less than 5 years (n = 8, 5.6%), 5–10 years (n = 41, 28.7%), 11–14 years (n = 63, 44.1%), and 15–18 years (n = 31, 21.7%). Regarding ethnicity, the largest group was Baloch (n = 43, 30.1%), followed by Sindhi (n = 35, 24.5%), Pashtuns (n = 24, 16.8%), Urdu-speaking (n = 16, 11.2%), Punjabi (n = 12, 8.4%), Saraiki (n = 8, 5.6%), Hindko (n = 4, 2.8%), and Kashmiri (n = 1, 0.7%). Majority i.e. 53.1% (n = 76) were underweight, 44% (n = 63) were within a healthy weight range while only 3 (2.1%) were overweight (85th to less than the 95th percentile) or obese (n = 1).(equal to or greater than the 95th percentile).

#### Clinical characteristics

Clinical characteristics of the total 143 patients are shown in Table 1. Bone or joint pain was the most frequent complaint (76.2%) that required analgesics’ intake (60.8%), history of fall (20.3%) and parental history of fracture (15.5%). Fractures in clavicle, humerus, and tibia were the most common. Blood transfusion rates varied, with the majority receiving 2 transfusions per month (66.4%). It is important to note that while majority of patients 93% (n = 133) were chelated for iron compliance with a major issue with regular chelation reported by 64.6% patients only. Noncompliance was also observed with oral calcium supplements and vitamin D supplements.

**Table 1:** Clinical characteristics of children with transfusion dependent beta thalassemia (n = 143).

Variables	Overall, N = 143	Males, n = 82	Females, n = 61
	n (%)	n (%)	n (%)
**Clinical History**			
Bone or joint pain	109 (76.2)	62 (75.6)	47 (77)
Pain requiring analgesics	87 (60.8)	52 (63.4)	35 (57.3)
Siblings with Beta Thalassemia	63 (44.1)	29 (35.3)	34 (55.7)
Prior history of falls	29 (20.3)	14 (17)	15 (24.5)
Breathlessness	28 (19.6)	18 (21.9)	10 (16.3)
History of weight loss	23 (15.5)	9 (10.9)	14 (22.9)
Parental history of fracture	23 (15.5)	8 (9.7)	15 (24.5)
Diarrhoea	11 (7.7)	6 (7.3)	5 (8.1)
**History of fracture**			
○ Location not specified	17 (11.9)	8 (9.75)	9(14.7)
○ Clavicle	4 (2.8)	2 (2.4)	2 (3.2)
○ Humerus	4 (2.8)	1 (1.2)	1 (1.6)
○ Tibia	2 (1.4)	1 (1.2)	1 (1.6)
○ Foot	2 (1.4)	1 (1.2)	1 (1.6)
○ Elbow	1 (0.7)	1 (1.2)	1 (1.6)
○ Femur	1 (0.7)	1 (1.2)	1 (1.6)
○ Humerus and elbow	1 (0.7)	1 (1.2)	1 (1.6)
○ Shoulder and hip	1 (0.7)	1 (1.2)	1 (1.6)
**Blood transfusions per month**			
○ 2/month	95 (66.4)	56 (68.2)	39 (63.9)
○ 1/month	32 (22.4)	12 (14.6)	20 (32.7)
○ 3-4/month	14 (9.7)	12 (14.6)	2 (3.2)
○ 5-8/month	2 (1.4)	2 (2.4)	-
**Drug History**			
Iron chelation	137 (93)	70 (85.3)	61 (100)
○ Oral Deferasirox	82 (57.3)	45 (54.8)	37 (60.6)
○ Oral Deferipone	27 (18.8)	20 (24.3)	7 (11.4)
○ Oral deferasirox+oral deferipone	22 (15.3)	4 (4.8)	18 (29.5)
○ Oral deferasirox+deferoxamine injections	1 (0.7)	1 (1.2)	-
○ Deferoxamine injections	1 (0.7)	-	1 (1.6)
Compliance with iron chelation			
• Regular	86 (64.6)	44 (53.6)	42 (68.8)
• Intermittent	47 (35.33)	22 (26.8)	25 (40.9)
• None	4 (3)	2 (2.4)	2 (3.2)
Oral calcium supplements	105 (73.4)	62 (75.6)	43 (70.5)
Oral vitamin D supplements	89 (62.2)	55 (67)	34 (55.7)
**Physical Examination**			
Splenomegaly	39 (25.7)	25 (30.4)	14 (22.9)
Prominent maxillary bones	26 (17.1)	15 (18.2)	10 (16.3)
Hepatomegaly	25 (16.4)	18 (21.9)	7 (11.4)
Limps when walking	18 (11.8)	9 (10.9)	9 (14.7)
Prominent zygomatic bones	15 (9.9)	11 (13.4)	3 (4.9)
Bowing of legs	6 (3.9)	4	2 (3.2)
Frontal bossing	1 (0.7)	-	1 (1.6)
**Dental Examination**			
Mild to moderate gingival inflammation	65 (45.4)	41(50)	24 (39.3)
Gingival pigmentation	57 (39.8)	36 (43.9)	21 (34.4)
Enamel hypocalcification	27 (18.8)	15 (18.2)	12 (19.6)
**Associated Health Disorders**			
Hepatitis C	6 (4.2)	4 (4.8)	2 (3.2)
Chronic hepatitis	4 (2.8)	3 (3.6)	1 (1.6)
Cardiomyopathy	3 (2.1)	1 (1.2)	2 (3.2)
Tuberculosis	2 (1.4)	2 (2.4)	-
Type 1 Diabetes	1 (0.7)	-	1 (1.6)
Growth hormone deficiency	1 (0.7)	1 (1.2)	-

### Biochemical markers

Hypocalcemia and hypophosphatemia were seen in 30.1% and 5.6%, respectively, while hypercalcemia and hyperphosphatemia were present in 0.7% and 3.5% of patients, respectively. Serum ferritin was significantly high, with a median of 2768.3 ng/mL (IQR = 2455.3). Vitamin D deficiency/insufficiency affected 92.3% of the participants in total while vitamin D toxicity (25OHD > 150 ng/ml) was not observed in any. Table 2 shows the overall and gender-wise distribution of the biochemical parameters tested. Females had higher median serum P levels (*p value* 0.0311) and lower median serum 25OHD levels (*p* value 0.0491) as compared to males. Additionally, females showed significantly higher serum ferritin levels (*p* value 0.0457), while males had higher median serum Cr levels (*p value* 0.0034). Females also exhibited a higher median TMP-GFR (*p* value 0.0204). Ca-P product was high in 14 (9.79%) of the participants. Parathormone deficiency was seen in 14%, while hyperparathyroidism was detected in 9.1% of patients.

**Table 2:** Overall and gender wise distribution of biochemical parameters of children with transfusion dependent beta thalassemia major.

Variables	Overall (n = 143)	Males (n = 82)	Females (n = 61)	*p*-value
**Haematological Parameters**	**Mean (±SD)**			
Haemoglobin (13.6–16.3 gm/dL)	8.2±1.15	8.1±1.1	8.4±1.2	0.11
Haematocrit (41.9–49%)	24.7±3.61	24.3±3.6	25.7±3.6	0.15
**Biochemical Parameters (Reference Range)**	**Median (Q3-Q1)**			
Serum Corrected Ca (8.6–10.2 mg/dl)	8.84 (0.6)	8.8 (0.58)	8.92 (0.5)	0.24
Serum P (4–7 mg/dl)	5 (1.0)	4.85 (0.9)	5.2 (1.0)	**0.0311**
Intact plasma PTH (16-87pg/ml)	38.4 (31.2)	37.2 (23.2)	39.3 (48.2)	0.82
Serum 25OHD (30–150 ng/ml)	16.1 (10/6)	17.25 (8.9)	13.6 (11.73)	**0.0491**
Serum 1, 25 (OHD)_2_ (19.9–70.3 pg/ml)	58.3 (32.4)	59.1 (32.7)	58.3 (32.4)	0.6
Serum Ferritin (7–140 ng/ml)	2768.3 (2455.3)	2472.35 (2570)	3181.8 (2174.3)	**0.0457**
Serum Cr (0.5–1 mg/dl)	0.3 (0.1)	0.4 (0.1)	0.3 (0.1)	**0.0034**
Serum Mg (1.6–2.6 mg/dl)	2 (0.3)	2 (0.3	2 (0.4)	0.5149
Plasma iFGF23 (cutoff 61.21 pg/ml)	38.18 (26.45)	37.98 (22.49)	39.4 (26.7)	0.3698
Plasma cFGF23 (33–145 RU/ml)	198.05 (355.56)	203.39 (370.58)	170.04 (276.5)	0.3897
**Urinary Indices**	**Median (Q3-Q1)**			***p* value**
TRP (85–95%)	97% (4)	96.8 (4.2)	97.3 (3.2)	0.3
TMP-GFR (2.8–4.4 mg/dl)	6.4 (1.69)	6.16 (1.6)	6.65 (1.2)	**0.0204**

Table 3 displays the median iFGF23 and cFGF23 levels associated with each of these biochemical diagnosis groups.

**Table 3:** Biochemical findings in relation to median FGF 23 levels in children with transfusion dependent beta thalassemia (n = 143).

Biochemical Parameter	Reference Ranges	n (%)	Median cFGF23 (IQR)	*p* value	Median iFGF23 (IQR)	*p* value
Serum Corrected Ca (mg/dl)	Normal 8.6–10.2	99 (69.23)	193 (406.4)	0.72	39.5 (24.8)	0.82
	Low <8.6	43 (30.07)	199.6 (333.1)		34.91 (29.3))	
	High >10.2	1 (0.7)	170.04 (0)		33.4 (0)	
Serum P (mg/dl)	Normal 4–7	130 (90.91)	192 (352.5)	0.53	39.2 (25.8)	0.1
	Low <4	8 (5.59)	286.2 (990.2)		16.9 (45.3)	
	High >7	5 (3.5)	134 (172.2)		19.3 (22)	
Intact plasma PTH (pg/ml)	Normal 16–87	110 (76.92)	183.7 (352.5)	0.74	38.2 (24.4)	0.5
	Low <16	20 (13.99)	169.0 (273.1)		42.8 (33.1)	
	High >87	13 (9.09)	340.2 (338.0)		35.5 (18.5)	
Serum 25OHD (ng/ml)	Normal 30–150	10 (6.99)	199.8 (805.3)	0.09	33.5 (36.7)	0.5
	Insufficiency 21–29.9	35 (24.48)	332.2 (566)		42.3 (27.2)	
	Deficiency <21	98 (68.53)	161.08 (250.5)		37.2 (25.9)	
Serum 1, (25OH)_2_D (pg/ml)	Normal 19.9–79.3	108 (75.52)	182.8 (258.4)	0.08	39.4 (23.1))	0.008*
	Low <19.9	7 (4.9)	1028.7 (782)		71.3 (35.5)	
	High >79.3	28 (19.58)	184.21 (402)		32.9 (32.6)	
Serum Ferritin (ng/ml)	High >322	143 (100)	198 (355.5)	-	38.2 (26.5)	-
Serum Cr (mg/dl)	Normal 0.5–1	24 (16.78)	479.1 (896.6)	0.1	45.24 (28.9)	0.09
	Low <0.5	118 (82.52)	170.8 (255.4)		36.5 (23)	
	High >1	1 (0.7)	472 (0)		132.3 (0)	
Serum Mg (mg/dl)	Normal 1.6–2.6	136 (95.1)	198.4 (434.7)	0.7	38.5 (25.7)	0.66
	Low <1.6	4 (2.8)	871.7 (1741.4)		34.2 (39.2)	
	High >2.6	3 (2.1)	155.8 (443.4)		28.1 (39.3)	
TMP-GFR (mg/dl)	Normal 2.8–4.4	2 (1.35)	647.8 (1875.5)	0.11	26.8 (52.8)	0.32
	Low <2.8	42 (27. 7)	842.2 (1542.2)		51.3 (4.4)	
	High >4.4	104 (70.2)	173 (271.9)		38.5 (22.9)	

Plasma iFGF23 was elevated (>61.21 pg/ml) in 14% (n = 29) while high plasma c-FGF23 (>145 RU/ml) was found in 60.8% (n = 87) of the participants. Low cFGF23 (33 RU/ml) was found in just 0.14% (n = 2) of the participants. People with low TMP-GFR (42 individuals, 27.7%) had higher median cFGF23 levels compared to those with high TMP-GFR (104 individuals, 70.2%), with values of 842.2 RU/ml and 173 RU/ml, respectively. Additionally, individuals with low TMP-GFR had higher median iFGF23 levels than those with high TMP-GFR, with values of 51.3 RU/ml and 38.5 RU/ml, respectively. Both cFGF23 and iFGF23 demonstrated an inverse relationship with serum ferritin levels, with rho coefficients of -0.2 and *p* value 0.01 for cFGF23 and -0.09 and *p* value 0.27 for iFGF23, respectively.

Serum P and c-FGF23 (rho coefficient = -0.21, *p* value 0.01*) showed an inverse correlation with each other. Serum ferritin levels showed a positive correlation with serum P (rho coefficient 0.17, *p* value 0.04*), as did TMP-GFR (rho coefficient 0.76, *p* value 0.000*). On univariate analysis, the primary exposure variable cFGF23, TMP:GFR, corrected calcium and serum ferritin were found to be significantly associated with Serum P with p-values of <0.25. The multivariable model explored these relationships collectively and is shown in Table 4. In the presence of significant interaction of corrected Ca and iPTH (p-value = 0.059), cFGF23, higher TMP:GFR levels showed a robust positive association, whereas corrected Ca had a significant negative association with Serum P. Plasma cFGF23 was kept in the model due to its position as the primary exposure variable, but serum ferritin became insignificant in the presence of other variables.

**Table 4:** Multivariate regression analysis of predictors of Serum P in children with transfusion dependent beta thalassemia (n = 143).

Serum P	Coefficient	95% Confidence Interval	Standard Error	t	P> |t|
cFGF23	-.0000423	-0.0001956, 0.000111	0.0000775	-0.55	0.586
TMP:GFR	0.42	0.3754895, 0.4750242	0.0251677	16.9	<0.001
Corrected Ca	-0.378054	-0.562913, -0.1931951	0.0934844	-4.04	<0.001
iPTH	-0.0246626	-0.0503455, 0.0010203	0.012988	-1.90	0.06
Interaction term of Corrected Ca and iPTH	0.0030523	-0.0001192, 0.0062237	.0016038	1.90	0.059

The table above provides information about each independent variable’s coefficient, standard error, t-value, p-value, and confidence interval. These values describe the relationships between the independent variables (cFGF23, TMP:GFR, Corrected Ca, iPTH, interaction term of Corrected Ca and iPTH) and the dependent variable (Serum P).

**Fig 1:**
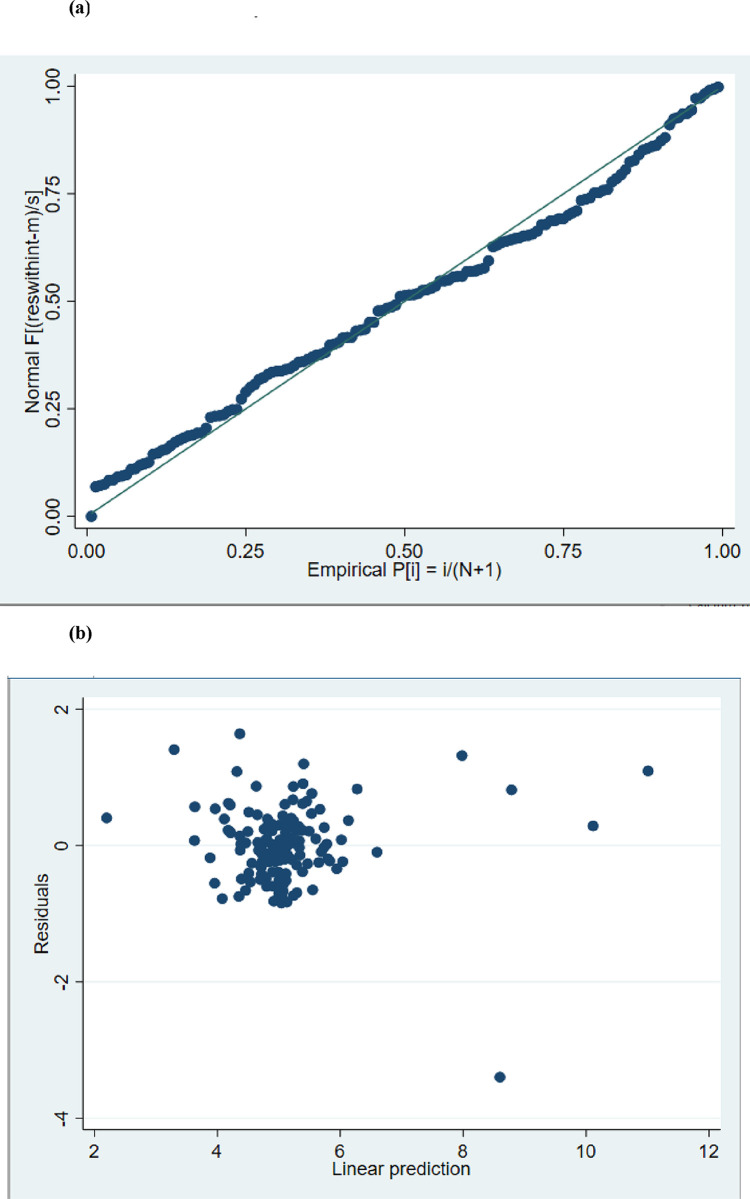
(a): Normal Probability Plot of Residuals Predicted in Multivariable Linear Regression Model of Serum P Associations. (b): Scatter Plot with Linear Prediction Showing Homoscedasticity of Residuals.

The multivariable model explained around 75% of the total variability in Serum P levels *(F = 85*.*25*, *p-value = <0*.*001*, *Adjusted R*^*2*^ *= 0*.*7479)*. Fig 1(A) and 1(B) display the residual plots of the predictors of Serum P identified in the multivariable regression model, which show an approximate agreement with normality.

## Discussion

The study findings highlight the associations between serum P and serum albumin-corrected Ca, renal function, c-FGF23, and iPTH. They provide insights into the complex interplay between P metabolism and other biomarkers in children with transfusion dependent β-TM. Numerous studies have been conducted to explore Ca-PTH-Vitamin D axis in thalassemia but one important unnoticed metabolic pathway in these patients is of the P homeostasis [14]. The influence of Fe overload in thalassemia on P metabolism and FGF23 concentration was assessed in the current study. The study examined the associations between serum P and various biochemical and clinical parameters through multiple linear regression and found interesting results. The three variables (excluding the individual variables in the interaction term) included in the multivariable model explain nearly 75% of the total variability in Serum P, indicating parsimony. The qualitative checks of goodness of fit and model adequacy depict adequacy, denoting that the model has a good set of predictors for Serum P in TDT patients.

The key aim of our research is to apply these findings to improve clinical management with potential to enhance outcomes by effectively preventing or managing phosphate disorders in patients with TDT. while our study does not establish specific cut-offs for biomarkers studied in TDT patients, it highlights important associations that may guide clinicians in identifying trends and risk factors related to hypo/ hyperphosphatemia, thereby informing more tailored management strategies. These findings underscore the meaningful relationships between these variables and serum P, highlighting their importance when analysing or predicting serum P levels. Studies investigating the relationship between PTH, serum P and Fe overload in children with thalassemia are few. Literature shows evidence of both hypo- [15, 16] and hyper-phosphatemia in patients with β-TM [5, 19]. Secondary hyperparathyroidism has also been observed as a common endocrine complication in these patients [18]. Overt hypoparathyroidism in thalassemia is relatively rare but asymptomatic hypoparathyroidism (up to 42%) has been observed [25]. Previous studies also showed that hypoparathyroidism was primarily associated with Fe overload in β-TM [26, 27], whereas other children and adults with thalassemia had relatively elevated serum P levels in many studies [28, 29].

High plasma iFGF23 levels were observed in 14% of the participants, while high c-FGF23 levels were observed in 60.8% in our study. Our data revealed that only 0.14% (n = 2) of the subjects had low cFGF23. cFGF23 and iFGF23 both had inverse relationship with serum ferritin levels in our data (rho coefficients -0.2 and -0.09, respectively). Study from Greece shows lower c-FGF23 levels in thalassemic patients (n = 40), higher iFGF23 levels in healthy controls (n = 41). Ferritin levels were inversely linked with serum c-FGF23 levels (r = -0,421, p = 0.018) [30]. Low P was found in 5.59% of the participants in the current study, with median cFGF23 levels of 286.2 RU/ml and iFGF23 levels of 16.9 pg/ml, respectively. While hyperphosphatemia was detected in 3.5%, with median cFGF23 levels of 134 RU/ml and iFGF23 values of 19.3 pg/ml, respectively [31]. Patients in their study had lower median plasma FGF-23 levels than controls [35.7 (2.1–242.8) vs. 53.2 (13.3–218.6) pg/mL]. The median FGF-23 level in hypoparathyroid patients was significantly lower than in normoparathyroid patients [34.8 (2.1–120.0) vs. 43.1 (3.2–242.8) pg/mL]. One mechanism by which Fe overload interferes with osteoid maturation/mineralization is the incorporation of Fe into calcium hydroxyapatite, which affects the growth of hydroxyapatite crystals and reduces bone unit tensile strength, affecting the dentition [15].

Iron overload in β-TM leads to reduced FGF-23 levels, which disrupts the normal P regulation mechanisms by increasing renal reabsorption of P with subsequent hyperphosphatemia. This dysregulation of P metabolism due to iron overload underscores the complex interplay between iron status, FGF-23, and P regulation. Limitations of the study include the fact that confounding factors like dietary intake and biological heterogeneity affecting P metabolism were not studied. Its cross-sectional design intends to generate baseline data of the factors studied.

## Recommendations

- Addressing barriers to treatment adherence for iron chelation therapy is crucial to ensure consistent therapeutic benefits and minimize the risk of iron overload complications in patients with β-TM. The prevalence of bone or joint pain among children with suggests the need for comprehensive pain management strategies to enhance patient comfort and quality of life.
- The high prevalence of underweight individuals underscores the importance of nutritional support interventions to address malnutrition and promote healthy growth and development in this population.
- The widespread occurrence of vitamin D deficiency highlights the necessity for routine screening with serum 25OHD, lifestyle modifications to ensure adequate exposure of sunlight to these children and/or Ca and Vitamin D supplementation to prevent bone and mineral and improve overall health outcomes.
- Elevated plasma levels of iFGF23 and c-FGF23 suggest potential disturbances in P metabolism in individuals with β-TM, necessitating further investigation into the underlying mechanisms and potential therapeutic interventions.
- Variations in P, along with differences in cFGF23 and iFGF23 levels based on TMP-GFR status, emphasize the complexity of P regulation in β-thalassemia major and underscore the need for personalized treatment approaches aimed at optimizing P homeostasis and improving patient outcomes. This may minimize the risk of related complications, particularly in individuals with varying levels of TMP-GFR.
- This study could serve as the basis for formulating guidelines to manage iron overload and its effects on phosphorus metabolism. Insights into the potential resistance to FGF23’s activities caused by iron overload may be especially valuable in treating complex disorders such as familial hypophosphatemia, which we want to establish through further longitudinal investigations.

## Conclusion

This study provides valuable insights into the complex interactions and metabolic pathways involved in P homeostasis in children with β-TM. In the presence of interaction between corrected Ca and iPTH, serum biochemical markers such as cFGF23 and TMP GFR were associated with disturbance in P metabolism in children with β-TM. Multidisciplinary approach involving chemical pathologists, haematologists, endocrinologists, metabolic bone specialists, and orthopaedic specialists is imperative for comprehensive bone care in such patients. Local guidelines are also needed for managing metabolic bone diseases in children with β-TM. For a comprehensive grasp of the mechanisms driving the disruption of P balance in iron-overloaded children with β-TM and for the formulation of targeted interventions, further exploration using a prospective cohort study design is crucial. This type of study would enable the longitudinal evaluation of iron overload, FGF-23 levels, and P regulation markers in a clearly defined group of individuals with β-TM. In-depth studies should explore the complex link between iron overload, FGF-23, and the Ca-P-PTH axis in thalassemia, investigating mechanisms, mineral metabolism, and clinical results. These studies will help in developing and validating guidelines and provide evidence-based recommendations for nutritional and pharmaceutical therapies to prevent P related metabolic bone disorders in β-TM [31]. This approach could provide important insights into the complex interactions between iron status, FGF-23, and phosphorus metabolism, enabling the development of targeted interventions to restore phosphorus balance in those affected.

## Supporting information

S1 Checklist STROBE statement—Checklist of items that should be included in reports of *cross-sectional studies*. (DOCX)

## Abbreviations

β-TM: Beta-thalassemia major
Fe: Iron
Ca: Calcium
P: Phosphate
25OHD: 25-hydroxyvitamin D
FGF23: Fibroblast growth factor 23
iPTH: Intact parathyroid hormone
CKD: Chronic kidney disease
AKU: Aga Khan University
FFK: Fatimid Foundation Karachi
HPLC: High-performance liquid chromatography
BMI: Body mass index
BSA: Body Surface Area
ERC: Ethical Review Committees
SPSS: Statistical Package for the Social Sciences
IQR: Interquartile range
USA: United States of America
ELISA: Enzyme-linked immunosorbent assay
TRP: Tubular maximum reabsorption of phosphate
eGFR: Estimated glomerular filtration rate
HPLC: High-performance liquid chromatography
Hb: Haemoglobin
HCT: Haematocrit
1,25OHD: 1,25-Dihydroxyvitamin D
Cr: Creatinine
Mg: Magnesium
iFGF23: Intact Fibroblast Growth Factor 23
cFGF23: C-Terminal Fibroblast Growth Factor 23
TMP-GFR: Tubular Maximum Reabsorption of Phosphate by Glomerular Filtration Rate
IQR: Interquartile Range
SD: Standard Deviation
